# Depression, anxiety, and post-traumatic stress symptoms among wildland firefighters and local residents in recurrent wildfire areas

**DOI:** 10.1186/s40359-026-05237-3

**Published:** 2026-07-25

**Authors:** Kanthee Anantapong, Thitiworn Choosong, Siriporn Thaiyapongsakul, Thammasin Ingviya, Udomsak Saengow

**Affiliations:** 1 Department of Psychiatry, Faculty of Medicine, Prince of Songkla University, Hat Yai, Thailand; 2 Department of Family Medicine and Preventive Medicine, Faculty of Medicine, Prince of Songkla University, Hat Yai, Songkhla 90110 Thailand; 3 Faculty of Medicine, Prince of Songkla University, Hat Yai, Thailand; 4 Department of Clinical Research and Medical Data Science, Faculty of Medicine, Prince of Songkla University, Hat Yai, Thailand; 5 School of Medicine, Walailak University, Nakhon Si Thammarat, Thailand; 6 Center of Excellence in Data Science for Health Study, Walailak University, Nakhon Si Thammarat, Thailand

**Keywords:** Disaster, Mental health, Emergency, Peat swamp

## Abstract

**Background:**

Recurrent wildfires are an emerging public health concern, yet little is known about their mental health impact on both wildland firefighters and local residents in low‑ and middle‑income countries. This study aimed to compare depression, anxiety, stress, and post‑traumatic stress symptoms between firefighters and residents living in a recurrent wildfire area in southern Thailand, and to identify factors associated with these outcomes.

**Methods:**

A cross‑sectional survey was conducted among 108 wildland firefighters and 1,090 residents in communities surrounding Kuan Kreng Peat Swamp. Mental health symptoms were assessed using the Depression Anxiety Stress Scales‑21 and the Post-Traumatic Stress Disorder (PTSD) Checklist for DSM‑5, and bivariate analyses plus multivariable logistic regression were used to examine associations with sociodemographic characteristics and wildfire experiences.

**Results:**

The prevalences of abnormal scores of depression, anxiety, stress, and PTSD symptoms were 14.8%, 21.8%, 5.9%, and 4.9%, respectively. The prevalences did not differ significantly between firefighters and residents. However, residents had higher median PTSD symptom scores, suggesting a greater subclinical PTSD burden. Experiencing actual or threatened death or serious injury was strongly associated with all four outcomes, with adjusted odds ratios of 2.68 for depression, 3.73 for anxiety, 3.13 for stress, and 8.20 for probable PTSD. A dose–response pattern was observed for wildfire‑related health concerns: compared with no concern, severe–extremely severe concern was associated with roughly two‑ to four‑fold higher adjusted odds of depression, anxiety, and stress, and more than five‑fold higher odds of probable PTSD. Shorter time since the most recent wildfire was linked to higher odds of depression and stress, and firefighters showed lower adjusted odds of depression than residents.

**Conclusions:**

Wildland firefighters and residents in this recurrent wildfire area showed similarly elevated prevalences of screen‑positive depression, anxiety, stress, and PTSD, although residents reported a greater overall PTSD symptom burden. Individuals with life‑threatening wildfire experiences, recent exposure, and intense wildfire‑related health concerns represent key priority groups for screening and psychosocial support, underscoring the need for integrated mental health strategies within wildfire preparedness and recovery efforts in resource‑limited settings.

## Background

Wildfires are increasingly common across the globe due to climate change and human-related ignitions, and they have significant health and economic impacts [1]. Recent estimates suggest that wildfire-related fine particulate matter may result in tens of billions of dollars in annual health-related economic damages by the end of this century, alongside increased mortality and cardiorespiratory morbidity [2–4].

Beyond direct physical harm, wildfires frequently cause displacement, family and relationship disruption, and interruption of work and study, contributing to prolonged social instability [5, 6]. Residents exposed to wildfires have higher rates of depression, anxiety, Post-Traumatic Stress Disorder (PTSD), sleep problems, and psychological distress compared with unexposed populations, and these effects can persist for years after the event [7, 8].

People living in areas affected by recurring wildfires are particularly vulnerable because repeated events and uncertainty about future fires can sustain psychological stress [6, 9]. Scoping reviews and recent population-based studies report that roughly 20–40% of wildfire-exposed adults experience clinically significant symptoms of depression, anxiety, or PTSD in the months after a severe wildfire [8, 10, 11], with prevalence estimates of moderate-to-severe depression, anxiety, and PTSD around 30%, 15–20%, and 10–20% in some communities [8].

Mental health problems are also common in wildland firefighters, who form the key frontline workforce during wildfire crises. They frequently experience long work hours, intense exposure to heat and smoke, and prolonged separation from family, often under temporary contracts that reflect limited recognition and support [12, 13]. Recent reviews and surveys report probable PTSD in approximately 10–30% of wildland firefighters, with depression ranging from about 14–44% and anxiety from 14 to 25% [7, 14–16].

Seasonal wildfires due to agricultural burning are common in Southeast Asia, including Thailand, Indonesia, and Malaysia, where forests and peatlands are sometimes illegally burned to clear land for cultivation [17]. These uncontrolled fires can become extensive wildfires, worsen regional haze, and create tensions and mistrust between communities, authorities, and wildland firefighting teams [18, 19].

Kuan Kreng Peat Swamp in Nakhon Si Thammarat Province, Thailand, our study site, covers approximately 5,600 hectares and has experienced recurrent peatland fires, with the highest number reported in 2019 (74 fire events, 15,435 Thai Rai or 2,470 hectares). Between 2018 and 2020, an estimated 51.68% of fires were attributed to deliberate burning and 9.4% to forest product collection, prompting the Department of National Parks, Wildlife and Plant Conservation (DNP), Ministry of Natural Resources and Environment, Thailand to implement wetland management, firebreak construction, awareness campaigns, and collaboration with local monitoring networks, although shortages of appropriate personal protective equipment persist [20].

To date, most research on wildfire-related mental health has been conducted in high-income countries, with limited evidence from low- and middle-income countries, particularly in Southeast Asia. Studies from Singapore have linked transboundary haze from Indonesian forest and peat fires to increased psychological symptoms, including anxiety, depressive symptoms, sleep disturbance, and stress or PTSD‑like intrusion and hyper‑arousal [21–23]. However, there is still very limited evidence on the mental health of people living directly in recurrent wildfire areas, and almost no research that directly compares wildland firefighters with residents in the same setting. Therefore, this study aimed to examine and compare depression, anxiety, and post-traumatic stress symptoms in wildland firefighters and residents repeatedly exposed to wildfire disasters in Kuan Kreng Peat Swamp, Thailand, to inform policies for mental health support in under-resourced fire protection systems in low- and middle-income countries.

## Methods

### Study design and setting

This study employed a cross-sectional design to assess mental health among peatland firefighters and nearby residents in the Khuan Khreng, an area with recurrent peatland fires. The Khuan Khreng peat swamp area located in Nakhorn Si Thammarat and Phatthalung Province, Thailand. However, the forest fire of the year 2024–2025 was mostly reported at the area of Cha-uat, Hua-sai, Chian Yai, Ron Phibun, and Chaloem Phra Kiat District of Nakhorn Si Thammarat Province.

### Participants

The study was conducted among two groups: (1) residents of the Khuan Khreng peat swamp area and (2) firefighters and wildfire prevention volunteers working in the area. Based on the 2003 National Survey showing an anxiety prevalence of 1.9% [24], the sample size was computed with α = 0.05, *p* = 0.019, a margin of error (d) of 0.01 to 0.05, and the population size (N) was 4320. The required total sample size was 615 to 1,723 participants.

### Sampling methods

Peatland firefighters and residents living in areas affected by swamp fires in Khuan Khreng were recruited using purposive sampling after the wildfire events. All eligible firefighters (*n* = 117) who were local residents and registered as former workers at Thale Noi Fire Control Station, Phru Khuan Kreng Fire Control Station, Thale Noi Non‑Hunting Area, or Bolo Non‑Hunting Area under the DNP were approached to participate. Data collection was initiated as soon as possible, typically within 1–3 months after official wildfire reports were issued by DNP. However, due to the geographic isolation of some villages and the need to obtain permission from village leaders, data collection in certain areas was delayed to 4–7 months. Residents were eligible if they had lived in and experienced wildfire events in the study area during 2024–2025. The sampling ratio of firefighters to residents (comparison group) was approximately 1:10.

### Inclusion criteria

Residents:

- Aged 20–60 years.
- Resided in an area affected by peatland fires for at least one year.

Firefighters:

- Aged 20–60 years.
- Local resident who has worked on peatland fire suppression for at least one year.

### Exclusion criteria

- Declined to participate.
- Had a documented history of psychiatric disorders diagnosed by a physician.
- Did not reside in Khuan Khreng Subdistrict.

## Materials

### Demographic and general health questionnaire

The self-administered questionnaire included items on demographics (gender, age, residence, marital status, religion, education, income, occupation, and medical history), occupational exposures (for wildfire firefighters only), wildfire experiences, and general health concerns related to wildfires.

### Depression Anxiety Stress Scales (DASS-21)

The DASS-21 is the abbreviated version of the original 42-item Depression Anxiety Stress Scales designed to measure symptoms of depression, anxiety, and stress as distinct but interrelated constructs. It consists of 21 items, seven per subscale, each rated on a four-point Likert scale ranging from 0 (“Did not apply to me at all”) to 3 (“Applied to me very much or most of the time”) [25]. Subscale scores are summed and multiplied by two to yield final scores consistent with the DASS-42 format. Cut-off scores for interpreting severity levels are as follows: for depression—normal (0–9), mild (10–13), moderate (14–20), severe (21–27), and extremely severe (≥ 28); for anxiety—normal (0–7), mild (8–9), moderate (10–14), severe (15–19), and extremely severe (≥ 20); and for stress—normal (0–14), mild (15–18), moderate (19–25), severe (26–33), and extremely severe (≥ 34) [26].

The Thai version of the DASS-21 has been extensively validated and demonstrates good reliability (Cronbach’s α: depression = 0.82, anxiety = 0.78, stress = 0.69) and robust psychometric validity [27]. The factor structure and interscale correlations align closely with the original version, supporting its use for population-based screening and research in Thai-speaking communities, particularly in occupational and environmental health studies [28].

### PTSD Checklist for DSM-5 (PCL-5)

The PTSD Checklist for DSM-5 (PCL-5) is a 20-item self-report measure assessing symptoms of post-traumatic stress disorder (PTSD) according to the Diagnostic and Statistical Manual of Mental Disorders, Fifth Edition (DSM-5) criteria [29]. Items correspond to the four symptom clusters: intrusion, avoidance, negative alterations in cognition and mood, and alterations in arousal and reactivity. Each item is rated on a five-point Likert scale from 0 (“Not at all”) to 4 (“Extremely”), reflecting symptom severity in the past month. Total scores range from 0 to 80, with higher scores indicating more severe PTSD symptoms. Suggested cut-off scores vary depending on population and study context, but a total score of 31 has been recommended as an optimal threshold for probable PTSD diagnosis in both clinical and general populations [30, 31].

The Thai version of the PCL-5 demonstrates excellent psychometric performance, with high internal consistency (Cronbach’s α = 0.96; subscale α = 0.86–0.91) and strong test–retest reliability (*r* = 0.82) [32]. These results indicate strong internal stability and temporal reliability, supporting its validity and cultural applicability for identifying PTSD symptoms among Thai adults exposed to traumatic or environmental stressors, including occupational hazards such as smoke exposure from fire events.

In this study, DASS‑21 and PCL‑5 cut‑offs were not used for clinical diagnosis, which would require formal clinical evaluation, but rather to classify participants as having screen‑positive symptoms or probable cases of depression, anxiety, stress, and PTSD. For brevity and readability, we occasionally refer to these outcomes as depression, anxiety, stress, and probable PTSD, while avoiding any implication of confirmed clinical diagnoses.

### Data collection and management

Data were collected using standardised paper-based questionnaires administered by trained research assistants to ensure consistency, accuracy, and participant confidentiality. Completed questionnaires were verified for completeness, assigned unique identification codes, and later entered into the KoboToolbox system using double data entry and random verification to minimise errors. All electronic data were stored in a password-protected, encrypted database accessible only to the principal investigator and authorized staff, with personal identifiers removed before analysis. Data were retained in accordance with institutional and national ethical standards and securely deleted after the required retention period.

### Data analysis

Following the statistical plan, data were coded using EpiData and analysed with R software. The Shapiro–Wilk test was used to determine the normality of the mental health status data, the dependent variables. While the independent variables included: sociodemographic data such as age, gender, marital status, smoking, alcohol consumption, traumatic experience, and lifestyle characteristics. Descriptive statistics were used to summarize participant characteristics, with categorical variables presented as frequencies and percentages, and continuous variables as means or medians, as appropriate. To compare mental health outcomes between firefighters and residents, independent sample t-tests or chi-square tests were conducted, depending on variable type. Logistic regression models were then applied to examine the association between mental health scores (DASS-21 and PCL-5) and potential explanatory factors such as demographic characteristics, occupational exposures, and smoking behaviour, adjusting for relevant covariates. Statistical significance was set at *p* < 0.05. Missing data were handled through listwise deletion (complete-case analysis) for descriptive analyses.

## Results

During 2024–2025, a total of 50 swamp fire events were recorded, affecting approximately 2,155 Thai Rai (or 345 hectares). Around 1,400 residents living in seven villages in Cha‑uat District, one village in Chaloem Phra Kiat District, and two villages in Ron Phibun District, Nakhon Si Thammarat Province were exposed to these events.

### Participant characteristics

In this study, 1198 participants were included, comprising 108 firefighters and 1090 residents (85.57% of respond rate). While the resident group had a relatively balanced gender distribution, almost all firefighters were male (91.7%; *p* < 0.001). The median age was 43 years in both groups, and marital status, religion, and duration of residence were similar, with most participants married, Buddhist, and having lived in the area for about 30–33 years. Firefighters were more likely than residents to have vocational or higher education (39.8% vs. 26.7%; *p* = 0.005) and to report incomes above the national average (43.4% vs. 12.3%; *p* < 0.001). Marked differences were seen in health behaviours: firefighters were more often daily smokers and less often non‑smokers than residents, and they had lower rates of alcohol abstinence (all *p* < 0.001) (Table 1).

**Table 1:** Participant characteristics (*N* = 1198)

Baseline characteristics	*N* (%)			Chi2 *P*-value
	Total *N* = 1198	Firefighter *N* = 108	Resident *N* = 1090	
Gender				< 0.001
Male	558 (46.6)	99 (91.7)	459 (42.1)	
Female	640 (53.4)	9 (8.3)	631 (57.9)	
Age (years)				0.651^a^
Median (IQR)	43 (32,51)	43 (33.8,50.2)	43 (32,51)	
Marital Status				0.545
Single	369 (30.8)	28 (26.2)	341 (31.3)	
Married	727 (60.7)	69 (64.5)	658 (60.4)	
Separated/ Divorced/ Widowed	101 (8.4)	10 (9.3)	91 (8.3)	
Education				0.005
High school and lower	864 (72.1)	65 (60.2)	799 (73.3)	
Vocational school, bachelor’s degree and higher	334 (27.9)	43 (39.8)	291 (26.7)	
Religion				0.978
Buddhism	1181 (98.6)	107 (99.1)	1074 (98.5)	
Islam	17 (1.4)	1 (0.9)	16 (1.5)	
Duration of residence in the area (years) (median, IQR)	32 (20,46)	30 (22.8,40)	33 (20,46)	0.549 ^a^
Monthly income (Baht)				< 0.001
Below national average	1005 (85)	60 (56.6)	945 (87.7)	
Above national average	178 (15)	46 (43.4)	132 (12.3)	
Smoking history				< 0.001
No	795 (66.4)	34 (31.5)	761 (69.8)	
Occasional smoker	97 (8.1)	6 (5.6)	91 (8.3)	
Daily smoker	275 (23)	59 (54.6)	216 (19.8)	
Ex-smoker	31 (2.6)	9 (8.3)	22 (2)	
Alcohol consumption				< 0.001
No	639 (53.4)	19 (17.8)	620 (56.9)	
Occasional drinker	434 (36.3)	68 (63.6)	366 (33.6)	
Daily drinker	95 (7.9)	9 (8.4)	86 (7.9)	
Ex-drinker	28 (2.3)	11 (10.3)	17 (1.6)	

^a^Mann-Whitney U test

Among the 108 firefighters, the median duration of service was 8 years (IQR 4–15). About half reported at least one episode per year of continuous exposure to wildfire smoke and heat lasting more than 24 h, while approximately one-third reported exposures of less than 8 h annually (Table 2).

**Table 2:** Working experience of firefighters (*N* = 108)

Demographic characteristics	*N* (%)
Duration of being a firefighter (years)	
Median (IQR)	8 (4,15)
Yearly average duration of continuous exposure to smoke and heat from wildfire	
Less than 8 h	33 (30.6)
8–16 h	9 (8.3)
17–24 h	4 (3.7)
More than a day	56 (51.9)
No answer	6 (5.6)

Most residents reported experiencing wildfires about once a year, whereas firefighters more often reported either no exposure or multiple events per year, suggesting different patterns or perceptions of exposure between occupational and community groups (Table 3). Firefighters generally reported fewer health concerns related to wildfires than residents, despite having a more recent median exposure (1 vs. 8 months), but were more likely to describe themselves as direct victims of events involving actual or threatened death or serious injury. Based on the PCL-5 cut-off of > 31, the overall prevalence of probable PTSD in this sample was 4.9%, and the prevalence of mild to extremely severe symptoms on the DASS-21 was 14.8% for depression, 21.8% for anxiety, and 5.9% for stress. The proportions of participants with probable PTSD and with abnormal DASS-21 subscale scores did not differ significantly between firefighters and residents; however, residents had a higher median PCL-5 score than firefighters, indicating a greater PTSD symptom burden at the symptom level despite similar categorical prevalence (Table 3).

**Table 3:** Traumatic experiences related to wildfires and mental health problems among study participants (*N* = 1198)

Traumatic experiences	*N* (%)			Chi2 *P*-value
	Total *N* = 1198	Firefighter *N* = 108	Resident *N* = 1090	
Ever experienced wildfire				< 0.001
No	98 (8.2)	31 (28.7)	67 (6.2)	
Once a year	818 (68.8)	36 (33.3)	782 (72.3)	
Twice a year and more often	273 (23)	41 (38)	232 (21.5)	
Time since the most recent wildfire (months)				< 0.001^a^
Median (IQR)	7 (6,10)	1 (1,3.5)	8 (6,10)	
Involved actual or threatened death and/or serious injury				0.099
Yes	86 (7.6)	12 (12.4)	74 (7.2)	
No	1044 (92.4)	85 (87.6)	959 (92.8)	
How the participant experienced the event				
Direct victim	131 (11.6)	47 (48.5)	84 (8.1)	< 0.001
Witness	734 (65)	10 (10.3)	724 (70.1)	< 0.001
Close family and friend	185 (16.4)	3 (3.1)	182 (17.6)	< 0.001
Repeatedly exposed as part of work	95 (8.4)	14 (14.4)	81 (7.8)	0.041
Others	12 (1.1)	8 (8.2)	4 (0.4)	< 0.001
Wildfire-related health concerns				< 0.001
No	415 (34.9)	80 (75.5)	335 (31)	
Mild - Moderate	621 (52.3)	25 (23.6)	596 (55.1)	
Severe - Extremely severe	152 (12.8)	1 (0.9)	151 (14)	
PCL-5 scores for PTSD				0.074
▪ Low PTSD (PCL-5 < = 31)	1097 (95.1)	107 (99.1)	990 (94.6)	
▪ Probable PTSD (PCL-5 > 31)	57 (4.9)	1 (0.9)	56 (5.4)	
▪ Median (IQR)	5 (0,14)	2.5 (0,8.2)	5 (0,15)	0.005^a^
DASS scores - Depression				0.523
▪ Normal	1017 (85.2)	94 (88.7)	923 (84.8)	
▪ Mild-moderate	155 (13)	10 (9.4)	145 (13.3)	
▪ Severe-extremely severe	22 (1.8)	2 (1.9)	20 (1.8)	
▪ Median (IQR)	0 (0,2.8)	0 (0,2)	0 (0,3)	0.199^a^
DASS scores - Anxiety				0.192
▪ Normal	934 (78.2)	90 (84.9)	844 (77.6)	
▪ Mild-moderate	195 (16.3)	13 (12.3)	182 (16.7)	
▪ Severe-extremely severe	65 (5.4)	3 (2.8)	62 (5.7)	
▪ Median (IQR)	1 (0,3)	0 (0,2)	1 (0,3)	0.062^a^
DASS scores - Stress				0.335
▪ Normal	1123 (94.1)	102 (96.2)	1021 (93.8)	
▪ Mild-moderate	55 (4.6)	2 (1.9)	53 (4.9)	
▪ Severe-extremely severe	16 (1.3)	2 (1.9)	14 (1.3)	
▪ Median (IQR)	1 (0,3.8)	1 (0,3)	1 (0,4)	0.769^a^

^a^Mann-Whitney U test

Across all four outcomes, sociodemographic and lifestyle characteristics (firefighter vs. villager, gender, education, income, smoking, alcohol use, and duration of residence) were broadly similar between participants with normal and abnormal scores on the DASS‑21 depression, anxiety, and stress subscales and the PCL‑5 (see Supplementary File 1 for bivariate analyses of all mental health disorders and associated factors). In contrast, older age, being married or previously married, greater health concerns related to wildfires, and exposure to life‑threatening or severe injury events showed consistent associations with all four symptom domains. Participants with abnormal depression scores tended to be slightly older and more often separated, divorced, or widowed, and were more likely to report severe health concerns related to wildfires and recent life‑threatening or severe injury events than those without depression, despite similar patterns of wildfire frequency and victim status. Abnormal anxiety scores were more common among older and women participants who were married or previously married and was strongly associated with wildfire‑related health concerns, life‑threatening or severe injury events, and repeated exposure as part of work (see Supplementary File 1 for bivariate analyses).

Abnormal stress scores were likewise more frequent among older, predominantly married participants and were linked to greater wildfire‑related health concerns, life‑threatening or severe injury experiences, and having close family members or friends affected by wildfires (see Supplementary File 1 for bivariate analyses). Probable PTSD cases were generally older, and more likely to be married, and had lower educational levels than those with normal PTSD scores. They were also more likely to report life‑threatening or severe injury events, witnessing wildfire‑related events, and moderate‑to‑severe health concerns related to wildfires, while distributions of gender, income, smoking, alcohol use, and frequency of wildfire exposure remained broadly comparable to participants with low PTSD scores (see Supplementary File 1 for bivariate analyses).

Table 4 presents multivariable models for probable depression, anxiety, stress, and PTSD, adjusted for group (resident vs. firefighter), gender, age, and covariates identified in bivariate analyses (see Supplementary File 1 for bivariate analyses). After adjustment, firefighters had significantly lower odds of abnormal depression scores than residents (adjusted OR = 0.42, 95% CI = 0.17–0.96), while group differences were not statistically significant for anxiety or stress, and PTSD could not be modelled by group because only one firefighter met the probable PTSD cut‑off.

**Table 4:** Factors associated with depression (*N* = 1107), anxiety (*N* = 1107), stress (*N* = 1106) and PTSD (*N* = 1111)

Factors	Depression			Anxiety			Stress			PTSD		
	Crude OR (95%CI)	Adjusted OR (95%CI)	*P*-value (LR-test)	Crude OR (95%CI)	Adjusted OR (95%CI)	*P*-value (LR-test)	Crude OR (95%CI)	Adjusted OR (95%CI)	*P*-value (LR-test)	Crude OR (95%CI)	Adjusted OR (95%CI)	*P*-value(LR-test)
Group			0.040			0.073			0.061			
Resident	Reference	Reference		Reference	Reference		Reference	Reference				
Firefighter	0.73 (0.35,1.38)	0.42 (0.17,0.96)		0.57 (0.29,1.02)	0.49 (0.22,1.07)		0.57 (0.14,1.58)	0.29 (0.06,1.05)				
Gender			0.723			0.151			0.805			0.748
Male	Reference	Reference		Reference	Reference		Reference	Reference		Reference	Reference	
Female	1.03 (0.74,1.44)	0.94 (0.66,1.34)		1.38 (1.03,1.86)	1.26 (0.92,1.75)		1.13 (0.68,1.9)	0.93 (0.54,1.65)		1.29 (0.74,2.32)	1.11 (0.6,2.09)	
Age (years)			0.318			0.016			0.096			0.530
Median (IQR)	1.01 (0.99,1.02)	1.01 (0.99,1.02)		1.02 (1.00,1.03)	1.02 (1.00,1.03)		1.02 (1.00,1.04)	1.02 (1.00,1.04)		1.03 (1.00,1.06)	1.01 (0.98,1.04)	
Marital Status												0.179
Single										Reference	Reference	
Married										3.03 (1.43,7.45)	2.33 (0.94,6.49)	
Separated/ Divorced/ Widowed										2.62 (0.76,8.4)	1.75 (0.45,6.44)	
Ever experienced wildfire												0.004
No / Once a year										Reference	Reference	
Twice a year and more often										0.47 (0.19,0.99)	0.33 (0.13,0.72)	
Time since the most recent wildfire (months)	0.94 (0.89,0.99)	0.91 (0.85,0.98)	0.006	0.97 (0.92,1.01)	0.94 (0.89,1.00)	0.050	0.89 (0.82,0.97)	0.85 (0.77,0.94)	0.001			
Involved actual or threatened death and/or serious injury			< 0.001			< 0.001			0.001			< 0.001
No	Reference	Reference		Reference	Reference		Reference	Reference		Reference	Reference	
Yes	3.48 (2.12,5.61)	2.68 (1.6,4.42)		4.54 (2.87,7.20)	3.73 (2.30,6.08)		4.85 (2.55,8.84)	3.13 (1.58,5.95)		10.19 (5.51,18.6)	8.2 (4.20,15.82)	
How the participant experienced the event												
Witness	0.7 (0.5,0.98)	0.7 (0.48,1.00)	0.052	0.74 (0.55,0.99)	0.71 (0.51,0.98)	0.04	0.64 (0.39,1.08)	0.66 (0.39,1.16)	0.149	0.56 (0.32,0.98)	0.63 (0.34,1.17)	0.142
Close family and friend							2.23 (1.24,3.85)	1.79 (0.97,3.20)	0.064			
Wildfire-related health concerns			0.003			< 0.001			0.004			< 0.001
No	Reference	Reference		Reference	Reference		Reference	Reference		Reference	Reference	
Mild - Moderate	2.04 (1.36,3.13)	1.88 (1.23,2.95)		2.63 (1.83,3.86)	2.32 (1.58,3.47)		3.24 (1.59,7.52)	2.76 (1.3,6.6)		4.28 (1.81,12.57)	3.8 (1.54,11.45)	
Severe - Extremely severe	2.41 (1.39,4.14)	2.37 (1.34,4.19)		3.28 (2.03,5.32)	2.98 (1.8,4.96)		4.57 (1.91,11.66)	4.46 (1.78,11.99)		7.65 (2.87,24.06)	7.53 (2.65,24.78)	

More frequent wildfire exposure (twice a year or more) was associated with lower odds of probable PTSD (adjusted OR = 0.33, 95%=CI 0.13–0.72), and shorter time since the most recent wildfire was associated with higher odds of abnormal depression and stress scores. Experiencing a life‑threatening or severely injurious wildfire was strongly associated with all four outcomes, with the highest adjusted odds for PTSD (adjusted OR = 8.20, 95% CI = 4.20-15.82). Wildfire‑related health concerns showed a clear dose–response pattern: compared with no concern, mild–moderate concern increased the odds of probable depression, anxiety, stress, and PTSD (e.g. adjusted OR = 2.32for anxiety and 3.80 for PTSD), and severe–extremely severe concern further elevated risk across all conditions (Table 4).

## Discussion

This study is, to the best of current knowledge, the first to examine mental health problems in both firefighters and residents living directly in a recurrent wildfire area, and one of the few conducted in a low‑ or middle‑income country context. A relatively high prevalence of screen-positive mental health problems was observed in both groups, ranging from 4.9% for probable PTSD to 21.8% for anxiety, with no statistically significant differences in prevalence between firefighters and residents. The identified associated factors can help characterise individuals who may be at increased risk of wildfire‑related mental health problems and inform public policies aimed at early detection and timely intervention.

Firefighters in this study generally had higher education levels and incomes than local residents. However, prior research has frequently reported underfunding of firefighters’ livelihoods, working conditions, and training, as well as inadequate income and reliance on temporary contracts [12, 33]—factors not specifically assessed here. Peatland fire management in Thailand is concentrated in a few southern peat swamps, particularly Kuan Kreng and Thale Noi, and is implemented by specialised forest fire and conservation units under national agencies. In Kuan Kreng, peat fires continue to recur despite coverage of all formal management stages because limited equipment and trained personnel leave volunteers at high risk, while difficult terrain, restricted access, complex fire behaviour, and low, unpaid volunteer engagement make suppression dangerous and often ineffective [20]. Consistent with this, the present study found poor working conditions among firefighter participants, including frequent 24‑hour continuous exposure to smoke and heat and experiences of life‑threatening or severe wildfire‑related injuries.

Residents and firefighters in this study showed broadly similar prevalences of clinically significant trauma and mood symptoms, consistent with ranges reported in previous wildfire research [8, 16]. However, residents reported more wildfire‑related health concerns and a heavier PTSD symptom load, reflected in higher median PCL‑5 scores. This pattern may be consistent with the possibility that cumulative exposure to fires, smoke, property threats, and chronic uncertainty sustains subclinical trauma symptoms among residents [6, 8, 34], whereas firefighters’ more occupationally framed exposure may be partly buffered by training, role identity, peer support, and a tendency toward lower risk perception reported in earlier studies [35]. Public concern may be mitigated by clear communication about smoke‑related cardiorespiratory risks [36, 37] and practical exposure‑reduction strategies, coordinated across land management, public health, and health services to protect high‑risk groups such as older adults, people with chronic heart or lung disease, and outdoor workers [38, 39].

Unexpectedly, this study found lower adjusted odds of probable depression among firefighters than residents and lower odds of probable PTSD among those reporting more frequent annual wildfire exposure. These results should be interpreted with particular caution, as they may reflect selection bias, survivor effects, exposure misclassification, or residual confounding rather than true resilience or habituation. This is consistent with prior work showing that repeated exposure to wildfires and other climate‑related disasters is generally associated with poorer mental health over time [40, 41], and helps avoid the “habituation fallacy,” whereby repeatedly affected communities are assumed to suffer less and consequently receive less support [42]. At the same time, the pattern may partly reflect greater preparedness and stress‑management training among firefighters, given that structured psychoeducational, peer‑support, and other psychological interventions can improve coping and reduce post‑traumatic symptoms in first responders [43].

Additionally, this study found that direct victims, witnesses, close relatives, and responders all reported PTSD, depression, and anxiety, underscoring the need to include both primary and indirectly or occupationally exposed groups in mental health response planning, in line with disaster PTSD reviews and guidance [44, 45]. Higher odds of screen-positive depression and stress closer to the most recent wildfire are consistent with evidence that symptoms often peak in the early post‑event period and then attenuate for some [8], while persistent and subthreshold problems remain common and clinically important [46]. The strong associations between actual or threatened death or serious injury and all four outcomes, together with the dose–response pattern for wildfire‑related health concerns, mirror prior work linking life threat, injury, and intense health worries with elevated PTSD, depression, and anxiety [47, 48], indicating that people with life‑threatening wildfire experiences and severe health concerns may warrant prioritisation for screening and targeted psychosocial support, especially where resources are limited.

### Strengths and limitations

This study was, to the best of current knowledge, the first to directly compare mental health problems between wildland firefighters and local residents living in areas with recurrent peat fires and included substantial numbers from both groups. However, several limitations should be noted. The cross‑sectional design precludes causal inference regarding wildfire exposure and mental health outcomes, and the average 7‑month interval since the most recent wildfire may have introduced recall bias and attenuated associations, as data collection did not coincide with peak wildfire periods.

Mental health outcomes were assessed using self‑report screening tools rather than clinical diagnostic interviews, so our findings reflect screen‑positive symptoms or probable cases rather than confirmed disorders. As both groups were recruited through purposive sampling, findings cannot be formally generalised beyond the defined study setting. Voluntary participation may also have introduced non-response bias, as those who declined may differ systematically from participants on key variables, including psychological distress, potentially affecting the observed associations.

The small firefighter sample, and the very limited number with probable PTSD, likely reduced the power and stability of between‑group comparisons. Although categorical prevalence was similar, residents had higher median PCL‑5 scores, indicating a greater PTSD symptom burden. The effect estimates for firefighters should therefore be interpreted with caution, as modest but clinically meaningful differences may have been missed. Some important potential confounders were not fully captured, including financial and social support, socioeconomic hardship, prior trauma, physical injury, displacement, property damage and home loss, histories of previous mental illness, resilience, and the content and intensity of firefighters’ training and fire exposure, which should be examined in future studies. Finally, the psychological consequences of wildfires are complex and dynamic, and qualitative research could further elucidate how wildfires affect local residents and wildland firefighters, as well as the interactions between these groups.

## Conclusion

This study showed that wildland firefighters and residents in a recurrent peat‑fire area had similar prevalences of depression, anxiety, stress, and probable PTSD, although residents carried a somewhat higher subclinical PTSD symptom burden. Life‑threatening or severe injury experiences, recent wildfire exposure, and intense wildfire‑related health concerns were strongly associated with all symptom domains, which may help identify priority groups for screening and psychosocial support in resource‑limited settings. The unexpectedly lower adjusted odds of depression among firefighters and of PTSD among those reporting more frequent wildfires should be interpreted cautiously, as they may reflect selection bias, survivor effects, exposure misclassification, residual confounding, or differences in preparedness, coping, or occupational support rather than indicating true resilience. These findings underscore the importance of developing and implementing mental health strategies that support both responders and residents in wildfire‑prone, low‑ and middle‑income settings.

## Data Availability

Data will be made available on request.

## Abbreviations

DASS-21: 21-item Depression Anxiety Stress Scales
DNP: Department of National Parks, Wildlife and Plant Conservation
DSM-5: Diagnostic and Statistical Manual of Mental Disorders, Fifth Edition
PCL-5: PTSD Checklist for DSM-5
PTSD: Post-Traumatic Stress Disorder
